# A discrimination model by machine learning to avoid gastrectomy for early gastric cancer

**DOI:** 10.1002/ags3.12714

**Published:** 2023-07-13

**Authors:** Tsutomu Hayashi, Ken Takasawa, Takaki Yoshikawa, Taiki Hashimoto, Shigeki Sekine, Takeyuki Wada, Yukinori Yamagata, Haruhisa Suzuki, Seiichirou Abe, Shigetaka Yoshinaga, Yutaka Saito, Nobuji Kouno, Ryuji Hamamoto

**Affiliations:** ^1^ Department of Gastric Surgery National Cancer Center Hospital Tokyo Japan; ^2^ Division of Medical AI Research and Development National Cancer Center Research Institute Tokyo Japan; ^3^ Cancer Translational Research Team, RIKEN Center for Advanced Intelligence Project Tokyo Japan; ^4^ Department of Diagnostic Pathology National Cancer Center Hospital Tokyo Japan; ^5^ Endoscopy Division National Cancer Center Hospital Tokyo Japan

**Keywords:** discrimination model, early gastric cancer, lymph node metastasis, machine learning, surgical indication

## Abstract

**Aim:**

Gastrectomy is recommended for patients with early gastric cancer (EGC) because the possibility of lymph node metastasis (LNM) cannot be completely denied. The aim of this study was to develop a discrimination model to select patients who do not require surgery using machine learning.

**Methods:**

Data from 382 patients who received gastrectomy for gastric cancer and who were diagnosed with pT1b were extracted for developing a discrimination model. For the validation of this discrimination model, data from 140 consecutive patients who underwent endoscopic resection followed by gastrectomy, with a diagnosis of pT1b EGC, were extracted. We applied XGBoost to develop a discrimination model for clinical and pathological variables. The performance of the discrimination model was evaluated based on the number of cases classified as true negatives for LNM, with no false negatives for LNM allowed.

**Results:**

Lymph node metastasis was observed in 95 patients (25%) in the development cohort and 11 patients (8%) in the validation cohort. The discrimination model was developed to identify 27 (7%) patients with no indications for additional surgery due to the prediction of an LNM‐negative status with no false negatives. In the validation cohort, 13 (9%) patients were identified as having no indications for additional surgery and no patients with LNM were classified into this group.

**Conclusion:**

The discrimination model using XGBoost algorithms could select patients with no risk of LNM from patients with pT1b EGC. This discrimination model was considered promising for clinical decision‐making in relation to patients with EGC.

## Graphical abstract

A discrimination model using machine leaning could select some pT1b tumors without LNM using the clinical data and pathological findings of the primary tumor.

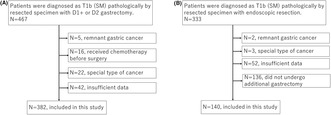

## 1 INTRODUCTION

In many cases, early gastric cancer (EGC) can be curatively treated with only local treatment. Endoscopic treatment is selected for cases in which lymph node metastasis (LNM) rarely develops, such as differentiated mucosal cancer (T1a) or small undifferentiated T1a. On the other hand, gastrectomy with regional nodal dissection is necessary when LNM is considered possible, such as submucosal cancer (T1b). However, only 15%–20% of T1b tumors have regional nodal metastasis.^1^, ^2^ LNM of gastric cancer rarely forms a visible mass; thus, LNM cannot be predicted based on imaging studies, such as multi‐slice computed tomography or endoscopic ultrasonography. LNM is only pathologically diagnosed after surgery.

Exploratory endoscopic treatment is another approach that may predict LNM more precisely using the pathological data of the primary tumor. Sekiguchi et al.^3^ reported a scoring system based on a multivariate logistic regression analysis using the data of surgically resected pT1a and pT1b. Hatta et al.^4^ also demonstrated another prediction model for the risk of LNM after endoscopic resection. Although these models^3^, ^4^ could predict a 1.6%–26.7% risk of LNM, these predictions are not clinically helpful to determine surgical indication because the possibility of LNM is not perfectly denied. As surgical mortality in patients with EGC is extremely rare, surgery is usually recommended even though the risk of LNM is only 2.5%.^4^

Different from classical statistical models, machine learning (ML), a computing system to increase accuracy by organizing algorithms for prediction, may be an attractive approach.^5^, ^6^, ^7^ Several studies have reported that ML could accurately predict LNM in colorectal,^8^ breast,^9^ thyroid,^10^ oral,^11^ and gastric^12^, ^13^ cancer. Moreover, the excellent diagnostic performance in predicting LNM of gastric cancer was confirmed in meta‐analysis.^14^ Recently, using clinical and pathological variables, Zhu et al.^15^ constructed a prediction model for LNM in patients with EGC using ML (eXtreme Gradient Boosting; XGBoost). Using their web‐based calculation model, physicians could calculate the precise risk of LNM. Although their model seems to have the best accuracy among previous reports, their prediction model is not clinically helpful for surgeons. Surgery is unavoidable as long as the possibility of LNM is not completely denied. What the physicians or patients want to know is not the risk of LNM but clinical question as to whether or not surgery is required.

In the present study, we attempted to construct a model using to select patients who do not require surgery for EGC.

## 2 METHODS

### 2.1 Study population

The development and validation cohorts were constructed using a prospectively collected pathological database of patients who received treatment at National Cancer Center Hospital, Tokyo between January 2013 and December 2018. For the development cohort, patients were selected according to the following criteria: initial gastrectomy with D1+ or D2 lymphadenectomy, pathological diagnosis of T1b, pathologically common type of adenocarcinoma, no remnant gastric cancer, and no chemotherapy before surgery. Using this development cohort, the discrimination model for predicting whether or not surgery is necessary (according to the prediction of LNM) was constructed by ML. Then, this discrimination model was validated in a different cohort (validation cohort), which was selected according to the following criteria: patient initially received endoscopic treatment for clinical T1a but was pathologically diagnosed with T1b, then received additional gastrectomy with D1+ or D2 lymphadenectomy, pathologically common type of adenocarcinoma, the horizontal and/or vertical margin of the resected specimen was not microscopically positive, and no remnant gastric cancer.

### 2.2 Clinicopathological evaluation

The extent of nodal dissection was determined according to the treatment guidelines of the Japanese Gastric Cancer Association (JGCA).^16^ Lymph nodes in each station were harvested immediately after surgery and fixed in formalin separated according to station. Then, LNM was evaluated pathologically. The primary tumor was also histologically examined in accordance with the recommendations of the Japanese classification of gastric carcinoma.

When patients initially received endoscopic treatment, the primary tumor was subjected to a more precise histological examination. In accordance with the JGGA guidelines, physicians recommended additional surgery when the tumor depth was pT1b, except in the case of differentiated tumors of <3 cm in diameter with a submucosal invasion depth of <500 μm without vascular invasion. This exception was based on the extremely low frequency of LNM.

### 2.3 Data preprocessing

The clinical data including patient's age, gender, and tumor location were retrieved from clinical database. The pathological findings including macroscopic type (elevated type, flat/depressed type, mixed type), histological type (pure differentiated type, pure undifferentiated type, mixed differentiated predominant type, mixed undifferentiated predominant type), ulcerative findings (present, absent), lymphatic invasion (present, absent), venous invasion (present, absent), and tumor size (long diameter, short diameter) were retrieved from pathological database. Furthermore, submucosal invasion depth and submucosal invasion size (long diameter, short diameter) were measured to add to the model variables.

Before developing the model, we performed data preprocessing for categorical, logical, and ordinal variables in the original data of both cohorts. First, the categorical and logical variables were converted to dummy variables with one‐hot encoding. Then, the ordinal variables (e.g., clinical and pathological staging) were converted to numeric variables. Finally, all “NA values” were filled with 0.

### 2.4 Establishment of the LNM prediction model

In this study, we used XGBoost as a ML algorithm, which has been reported to be the best model for predicting LNM of several malignancies in the previous studies.^15^ First, we developed the LNM prediction model using the dataset of the development cohort. The discriminative abilities of the model were evaluated by calculating the area under the receiver operating characteristic curve (AUC). To tune the hyperparameter, we performed 10‐fold cross‐validation 100 times with randomized parameters and development‐validation sets. Furthermore, the development dataset was applied to the over‐sampling algorithm, SMOTE (10.1613/jair.953), to overcome class imbalance. Then, we used the AUC as the tuning metric and obtained the best parameter to maximize the metric. Lastly, we generated the final prediction model with the tuned hyperparameter and applied the developmental dataset. Then, we plotted the receiver operating characteristic curve (ROC) of the predicted results.

### 2.5 Assessment of the discrimination model

The goal of the discrimination model is to select the patients who do not require additional surgery without selecting even a single patient with LNM. The performance of the discrimination model was evaluated based on the number of cases identified as having no indications for additional surgery with the prediction of LNM as true negatives, with no false negatives allowed. The classification threshold set the value when all cases with LNM in the development dataset were diagnosed as “LNM positive.” This threshold was identified as the probability value that was calculated by the LNM prediction model at the recall value for the prediction of possible LNM positivity becomes 1.0 in the development cohort. The classification of LNM negativity was determined when the probability value calculated by the LNM prediction model was below this threshold probability value. The same probability value was used as a threshold for classification in the validation cohort.

### 2.6 Feature importance of the LNM prediction model

The feature importance of the XGBoost model was calculated by the averaged gain of the features of each tree using xgb.importance, which is a function of the R package XGBoost (1.5.0.2).

### 2.7 Software and packages

All computational procedures were performed with R (4.1.1) and the following R packages: tidyverse (1.3.1), magrittr (2.0.2), caret (6.0‐90), XGBoost (1.5.0.2), Epi (2.44).

### 2.8 Ethical considerations

This study was approved by the Institutional Review Board of the National Cancer Center (2022 epidemiologic study‐200). Informed consent to be included in the study, or the equivalent, was obtained from all patients.

## 3 RESULTS

### 3.1 Baseline characteristics

**FIGURE 1:**
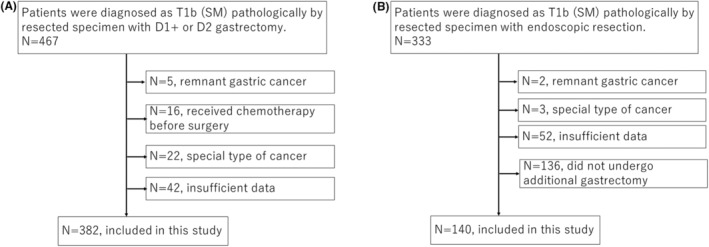
Consort diagram of included patients. (A) Development cohort. (B) Validation cohort.

Figure 1A shows the consort diagram of the development cohort. Forty‐two were excluded due to insufficient data patients. Then, a total of 382 patients were entered into the development cohort. Figure 1B shows the consort diagram of the validation cohort. One hundred and thirty‐six patients refused to undergo additional gastrectomy and were excluded. Finally, 140 patients were included in the validation cohort.

The baseline characteristics of patients in the development and validation cohorts are summarized in Table 1. LNM was observed in 95 patients (25%) in the development cohort and 11 patients (8%) in the validation cohort. In the validation cohort, the incidence of LNM (8%) was lower than that of the development cohort (25%). The development cohort had a larger tumor size, deeper submocosal (SM) invasion depth, more undifferentiated tumors, and more lymphovascular invasion in comparison to the validation cohort, indicating that the development cohort included more advanced cases.

**TABLE 1:** Clinicopathological characteristics.

Characteristics	Development cohort (*n* = 382)	Validation cohort (*n* = 140)
Age, median (range)	67 years (35–91)	71 years (43–89)
Gender		
Male	246 (64%)	105 (75%)
Female	136 (36%)	35 (25%)
Tumor size, median (range)	30 mm (3–130)	17.5 mm (3–52)
Tumor location		
Gastroesophageal junction	15 (4%)	0 (0%)
Upper third	77 (20%)	41 (29%)
Middle third	171 (45%)	48 (34%)
Lower third	119 (31%)	51 (37%)
Macroscopic type		
Elevated	31 (8%)	17 (12%)
Flat/depressed	253 (66%)	106 (76%)
Mixed	98 (26%)	17 (12%)
Depth		
SM1	83 (22%)	46 (33%)
SM2	299 (78%)	94 (67%)
SM invasion depth, median (range)	1225 μm (40–20 000)	655 μm (30–5500)
SM invasion size, median (range)	10 mm (0.1–52)	5 mm (0.1–27)
Histological type		
Pure differentiated type	146 (38%)	106 (77%)
Pure undifferentiated type	124 (33%)	9 (6%)
Mixed type, differentiated predominant	76 (20%)	16 (11%)
Mixed type, undifferentiated predominant	36 (9%)	9 (6%)
Ulcerative findings		
Present	129 (34%)	14 (10%)
Absent	253 (66%)	126 (90%)
Lymphovascular invasion		
Present	106 (72%)	65 (46%)
Absent	276 (28%)	75 (54%)
Venous invasion		
Present	139 (37%)	47 (34%)
Absent	243 (63%)	93 (66%)
Lymph node metastasis		
Present	95 (25%)	11 (8%)
Absent	287 (75%)	129 (92%)

### 3.2 Performance of the LNM prediction model

**FIGURE 2:**
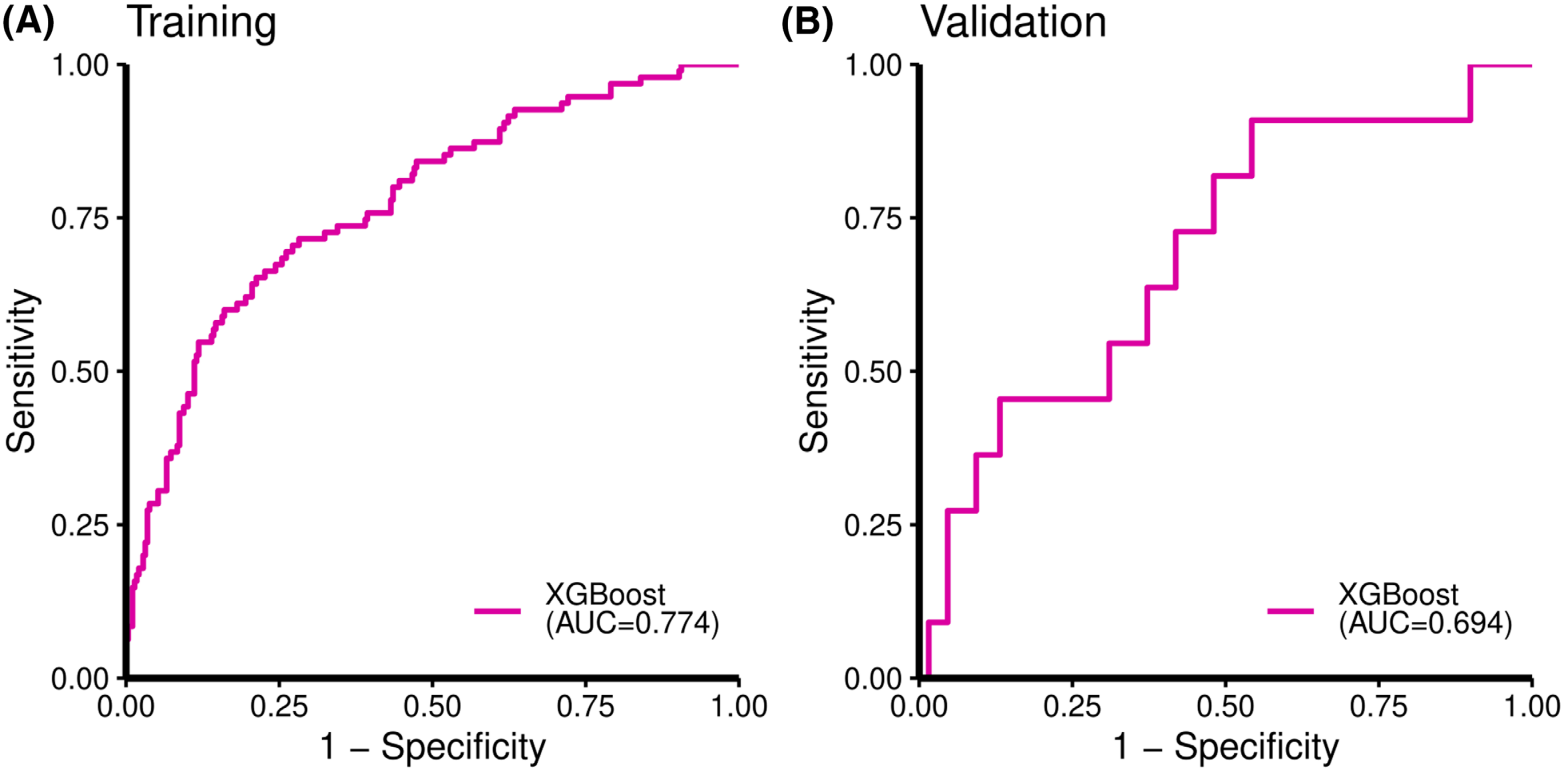
The receiver operating characteristics curves of this lymph node metastasis prediction model. (A) Development cohort. (B) Validation cohort.

The receiver operating characteristics curves of this LNM prediction model are shown in Figure 2. The area under the curve (AUC) was 0.774 in the development cohort and 0.694 in the validation cohort. Table 2 shows the performance metrics of both cohorts when Youden's J statistic,^17^ which is calculated by subtracting 1 from the sum of sensitivity and specificity, is the maximized. Both the development and validation cohorts showed high specificity, 0.787 and 0.860, respectively. The balanced accuracy, which is an evaluation index in the unbalanced binary classification, was 0.720 and 0.657, respectively. The differences in sensitivity, precision, and F1 between the development and validation cohorts were thought to be due to differences in the proportion of LNM positives in each cohort.

**TABLE 2:** Performance of prediction model for development and validation dataset.

	Sensitivity	Specificity	Precision	F1	Balanced accuracy
Development	0.652631579	0.787456446	0.504065041	0.568807339	0.720044012
Validation	0.454545455	0.860465116	0.217391304	0.294117647	0.657505285

### 3.3 Feature importance

**FIGURE 3:**
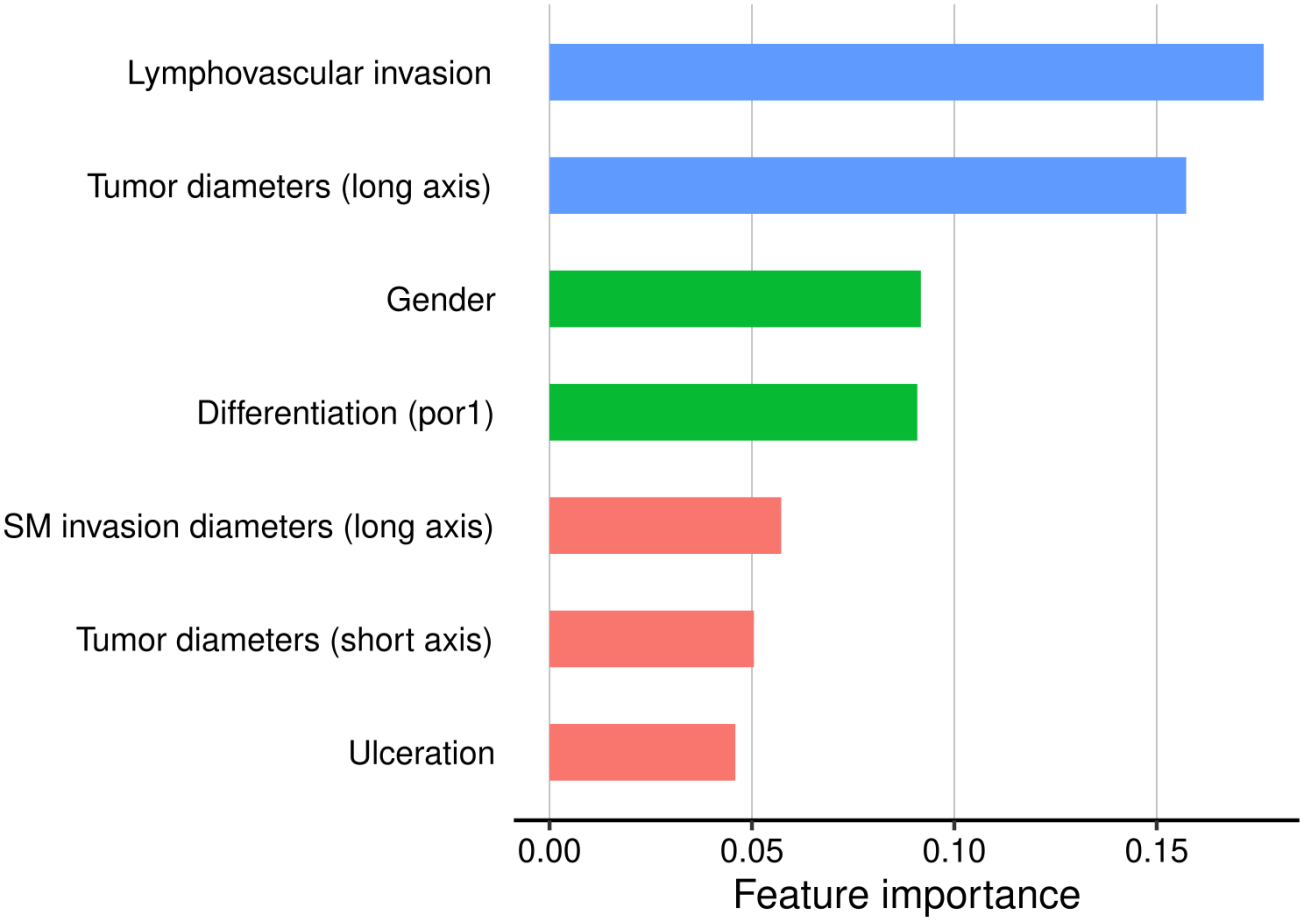
Relative importance of variable for predicting lymph node metastasis as positive in XGBoost.

The relative importance of each variable in this model is shown in Figure 3. Lymphatic invasion showed the highest importance, followed by tumor diameter, gender, poor differentiated histological type, distance of submucosal invasion, and presence of ulceration.

### 3.4 Discrimination performance not allowing false negatives for LNM

**FIGURE 4:**
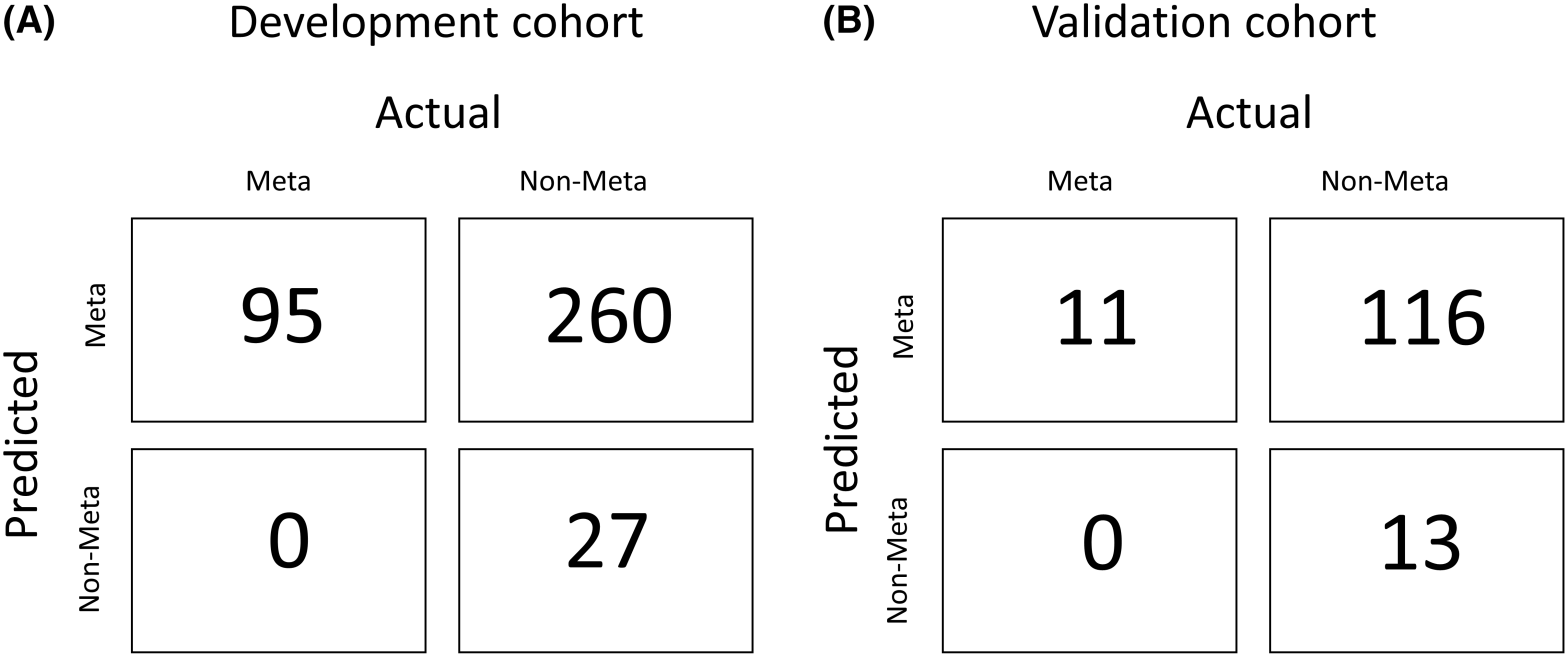
The confusion matrix representing the correct and incorrect predictions of the models and the actual lymph node metastasis stats in development cohort (A), and validation cohort (B).

We set a threshold for no surgery under the condition that the probability value was 0.160575 where the recall value for the prediction of possible LNM positivity becomes 1.0. Figure 4A shows the confusion matrix of the development cohort, representing the information of the correct and incorrect predictions of the models and the actual LNM status. Among 382 patients in the development cohort, 27 were accurately diagnosed as LNM‐negative by this LNM prediction model. Figure 4B demonstrates the confusion matrix of the validation cohort, predicting LNM with the same prediction threshold used in the development cohort. Among the 140 patients of the validation cohort, 13 patients could be accurately selected as LNM‐negative by this model. The performance of this prediction model in the development and validation cohorts is shown in Table 2.

### 3.5 Characteristics of patients identified as having no indications for additional surgery by XGBoost in the validation cohort

Table 3 showed details of the patients who were identified as having no indications for additional surgery by this discrimination model. For each patient, we also calculated the risk of LNM based on the Sekiguchi model.^3^ Only one patient was judged to have no risk (0%) of LNM. In the other 12 patients, the risk of LNM ranged from 2.0% to 30%.

**TABLE 3:** Detail of the patients who were discriminated as lymph node metastasis negative in our model.

Case	Age	Gender	Location	Macroscopic type	Tumor size (mm)	SM invasion size (mm)	SM invasion depth (μm)	Histological type	Ly^a^	v^b^	UL^c^	Predict value Sekiguchi model (%)
1	73	Female	M	IIa	21	1	280	tub1	−	+	−	6.7
2	63	Female	L	IIa + IIc	33	1	180	tub1	−	−	−	2.0
3	71	Female	M	IIc	21	5	270	por2, sig	−	−	−	3.8
4	73	Female	M	IIc	3	1	300	tub1	−	+	−	7.5
5	68	Female	M	IIc	6	0.5	400	tub1	−	+	−	7.5
6	82	Female	L	IIa + IIc	12	1	350	tub1	−	−	−	0.0
7	68	Female	M	IIa	20	4	600	tub1	−	−	−	2.0
8	73	Female	U	IIc	25	1.6	510	tub1	−	−	−	3.8
9	63	Female	U	IIa	21	3	550	tub2, por2	−	+	−	30.0
10	68	Female	M	IIc	12	3	600	por2, sig	−	−	−	7.5
11	71	Female	U	IIc	15	2	550	tub1, pap	−	−	−	2.0
12	77	Female	M	IIc	11	3	900	tub1	−	−	−	2.0
13	61	Female	U	IIa + IIc	15	10	3500	tub1	−	−	−	2.0

^a^ Lymphatic invasion.

^b^ Vascular invasion.

^c^ Ulcerative findings.

## 4 DISCUSSION

Machine learning has been reported to predict LNM with high accuracy, but it has not been established how to utilize ML for clinical practice. To accurately select the patients who do not require surgery for pT1b gastric cancer, we constructed a discrimination model by ML in the development cohort, which included patients with a risk of LNM, then validated its usability in the validation cohort, which included patients for whom additional surgery was required. In the development cohort, this discrimination model correctly selected 27 patients among 287 who had no pathological LNM and proposed no surgery for these 27 patients among 382 patients who were diagnosed with pT1b without any false negatives for LNM. Among 140 patients in the validation cohort, this discrimination model accurately selected 13 patients who have no risk of LNM without any false negatives for LNM. These results suggest that with this prediction model that was developed using ML, additional surgery could be avoided in almost 10% of patients who received endoscopic treatment and were subsequently diagnosed with pT1b. This has never been achieved using conventional statistical prediction of LNM.

Among 140 patients in the validation cohort of the present study, only one patient was accurately predicted to be LNM‐negative by the Sekiguchi model, while 13 were accurately predicted by our prediction model. When looking at the details of 13 patients in Table 3, tumors with undifferentiated histology or with deep submucosal invasion were selected. This prediction model could select such tumors in which the risk of LNM had never been ignored. The conventional statistical LNM prediction models were based on the nomogram developed using a multivariate logistic regression analysis, which follows the assumption that each explanatory variable is independent for one target and that the relationship between both variables is linear. However, variables are not always independent, and the relationships between variables do not always fit a linear model. Among data for predicting specific indicators, ML algorithms can handle many different types of variables, even with complex internal relationships. For example, the short and long axis of the tumor are almost proportional. Bigger tumors tend to invade deeper. Venous invasion is frequently observed in tumors that have lymphatic invasion. Accordingly, ML can be a powerful tool when the variables are clinical and pathological data.

Zhu et al.^15^ constructed an excellent model to predict LNM in EGC by the XGBoost algorithm, using clinical and pathological findings of endoscopically resected primary tumors. Their prediction model showed high accuracy in the prediction of LNM with an AUC of 0.827, indicating that ML, especially XGBoost, was suitable for the prediction of LNM in EGC after endoscopic resection. The AUC of our prediction model was 0.777, which was slightly inferior to that of the model developed by Zhu et al., which would be due to differences in the cohort. The prediction model developed by Zhu et al. included pT1a and pT1b gastric cancer, whereas our model targeted only pT1b. As the frequency of LNM is extremely low in pT1a, it is not surprising to see a very high AUC in the model developed by Zhu et al. On the other hand, the AUC in the validation cohort was slightly inferior to that of the development cohort.

For the development of the discrimination model, we selected patients who underwent initial surgery with lymph node dissection without any prior treatment for the primary tumor. After that, we evaluated this discrimination model using patients who underwent additional surgery after endoscopic resection of the primary tumor. Although the patients included in both cohorts were pathologically diagnosed with T1b EGC, the basal characteristics of the primary tumor were different, as shown in the results. This difference is explained by the different indications for initial treatment depending on the clinical stage. In the case of clinical diagnoses, such as T1a with differentiated histology—in which LNM rarely develops—physicians initially select endoscopic treatment. When the risk of LNM is not zero (e.g., clinically apparent T1b with undifferentiated histology), physicians select initial surgery. As a result, the rate of LNM was higher in the development cohort than in the validation cohort. Thus, the discrimination model that was constructed in the development cohort would be validated in a cohort in which the risk of LNM is low and prediction is difficult.

In our prediction model for LNM, lymphatic invasion was the most critical variable for predicting LNM, followed by tumor diameter, gender, poorly differentiated histology, and submucosal invasion diameter. Lymphatic invasion, tumor size, and poorly differentiated histology are well‐known risk factors for LNM.^1^, ^3^, ^4^, ^18^ In particular, lymphatic invasion has been reported to be the strongest predictor of LNM,^3^, ^4^ which was in line with our results. Although not much is known about the significance of gender with regard to the development of LNM, endogenous estrogen reportedly plays a role in the LNM of EGC.^19^ However, it is unclear whether that finding had any impact on our results of tendency to LNM. We cannot rule out the possibility that the limited number of cohorts led to such a result by chance. As we considered that the volume of submucosa invaded by the tumor strongly influenced LNM, we evaluated the invasion size and depth of the submucosa and included these values in the prediction model. However, invasion depth of the submucosa was the fifth most critical variable. Regarding the size of the tumor, evaluating the mucosal surface was found to be a stronger determinant of LNM than the submucosal area.

The present discrimination model has some issues to overcome for utilization in clinical practice. First, the LNM is pathologically diagnosed by one representative slice of resected lymph nodes in general, which was also the same in the present study. Thus, pathological N0 includes micro‐metastasis which cannot be identified with one representative slice. Such micro‐metastasis might be only curable by surgical resection. Therefore, it is essential to confirm whether no surgery based on our prediction model has similarly high survival as compared with the current standard surgery. Second, in the present model, about 10% of validation cohort were selected as negative for LNM, which is still unsatisfactory considering the actual rate of LNM in pT1b gastric cancer. To improve the prediction, the image recognition of resected specimen using deep learning instead of textual pathological data might be a key approach. Finally, as undifferentiated tumors are likely to metastasize to lymph node, the prediction of LNM for undifferentiated tumors might be difficult even by ML. However, three patients with undifferentiated type were chosen to be N0 in validation cohort in the current model. Although the number of predictive N0 in the undifferentiated type was not so much greater than that of differentiated type, there is a chance to predict N0 even in the undifferentiated tumors using the current model. Further improvement of the prediction would be necessary.

The present study was associated with several limitations. First, our discrimination model was developed and validated in a prospectively collected database; however, the treatments were not prospectively determined. The extent of dissection could be limited depending on the risk of the patients. A prospective study is required to confirm the usability of our discrimination model. Second, the manner of the pathological evaluation differed between the development and validation cohorts. For the pathological examination, the primary tumor is cut every 5 mm in a surgically resected specimen, while it is cut every 2 mm in an endoscopically resected specimen. By examining the resected specimen in more detail, factors involved in LNM, such as lymphovascular invasion and submucosal invasion area could be more likely to be detected. Third, the surgically resected specimen was pathologically evaluated only by routine hematoxylin–eosin staining. The addition of immunostaining, including D2‐40 staining, could contribute to increasing the detection rate of lymphatic invasion. The present discrimination model might be improved by additional immunostaining observations. Fourth, our discrimination model was validated in a dataset of patients who underwent additional surgery after endoscopic resection. In this validation cohort, only about half underwent additional surgery, which could have caused a selection bias. We strictly had followed the Gastric Cancer Treatment Guideline on the indication for endoscopic resection and additional surgery after endoscopic resection. For the patients who required additional surgery after endscopic resection (ER), the physicians had explained the risk of nodal metastasis, the necessity of additional surgery, and the risk of recurrence without additional surgery, without special intention. However, nearly half of the patients indicated for additional surgery had rejected our proposal. The reason why the patients refused additional surgery is unclear. Finally, the sample size was 382 patients in the development cohort and 140 patients in the validation cohort, which would have limited its reliability.

In conclusion, a discrimination model using XGBoost algorithms have potential to select some pT1b tumors without LNM (without any false negatives for LNM) using the clinical data and pathological findings of the primary tumor. With this discrimination model, some patients could avoid additional gastrectomy after exploratory endoscopic treatment. To validate this discrimination of reliability in clinical practice, prospective survival analysis was required in future.

## AUTHOR CONTRIBUTIONS

TH, KT, and TY are responsible for the study concept, data collection, and writing the article. KT is responsible for developing and evaluating model. The other authors collected data, reviewed and corrected the article. The authors read and approved the article.

## FUNDING INFORMATION

No funding.

## CONFLICT OF INTEREST STATEMENT

The authors declare no conflicts of interest for this article.

## ETHICS STATEMENTS

Approval of the research protocol: All procedures followed were in accordance with the ethical standards of the responsible committee on human experimentation (institutional and national) and with the Helsinki Declaration of 1964 and later versions.

Informed consent: Informed consent to be included in the study, or the equivalent, was obtained from all patients.

Registry and the Registration No. of the study/trial: N/A.

Animal Studies: N/A.
